# Neurodevelopmental disorders and subsequent risk of violent victimization: exploring sex differences and mechanisms

**DOI:** 10.1017/S0033291721003093

**Published:** 2021-09-01

**Authors:** Laura Ghirardi, Ralf Kuja-Halkola, Erik Pettersson, Amir Sariaslan, Louise Arseneault, Seena Fazel, Brian M. D’Onofrio, Paul Lichtenstein, Henrik Larsson

**Affiliations:** 1 Department of Medical Epidemiology and Biostatistics, Karolinska Institutet, Stockholm, Sweden; 2 Faculty of Social Sciences, Social and Public Policy Unit, University of Helsinki, Helsinki, Finland; 3 Social, Genetic, and Developmental Psychiatry Centre, Institute of Psychiatry, Psychology, and Neuroscience, King’s College London, London, United Kingdom; 4 Department of Psychiatry, University of Oxford, Warneford Hospital, Oxford, United Kingdom; 5 Department of Psychological and Brain Sciences, Indiana University, Bloomington, Indiana; 6 School of Medical Sciences, Örebro University, Örebro, Sweden

## Abstract

**Background:**

Neurodevelopmental disorders (NDs) are associated with experiences of victimization, but mechanisms remain unclear. We explored sex differences and the role of familial factors and externalizing problems in the association between several NDs and violent victimization in adolescence and young adulthood.

**Methods:**

Individuals born in Sweden 1985-1997, residing in Sweden at their 15^th^ birthday, were followed until date of violent victimization causing a hospital visit or death, death due to other causes, emigration, or December 31^st^ 2013, whichever came first. The exposures were diagnoses of attention-deficit/hyperactivity disorder (ADHD), autism spectrum disorder (ASD), intellectual disability (ID) and other NDs. We used three different Cox regression models: a crude model, a model adjusted for familial confounding using sibling-comparisons, and a model additionally adjusted for externalizing problems.

**Results:**

Among 1,344,944 individuals followed, on average, for 5 years, 74,487 were diagnosed with NDs and 37,765 had a hospital visit or died due to violence. ADHD was associated with an increased risk of violent victimization in males (HR=2.56; 95% CI=2.43-2.70) and females (HR=5.39; 95% CI=4.97-5.85). ASD and ID were associated with an increased risk of violent victimization in females only. After adjusting for familial factors and externalizing problems, only ADHD was associated with violent victimization among males (HR=1.27; 95% CI=1.06-1.51) and females (HR=1.69; 95% CI=1.21-2.36).

**Conclusions:**

Females with NDs and males with ADHD are at greater risk of being victim of severe violence during adolescence and young adulthood. Relevant mechanisms include shared familial liability and externalizing problems. ADHD may be independently associated with violent victimization.

## Introduction

Individuals with neurodevelopmental disorders (NDs), such as autism spectrum disorder (ASD), attention-deficit/hyperactivity disorder (ADHD), and intellectual disability (ID) (APA, 2013), are at increased risk of being victim of violence. For example, there is evidence that ADHD is associated with an increased risk of physical (Dammeyer & Chapman, 2018), sexual (Dammeyer & Chapman, 2018; Ohlsson Gotby, Lichtenstein, Langstrom, & Pettersson, 2018), dating (McCauley et al., 2015), and intimate partner victimization (Guendelman, Ahmad, Meza, Owens, & Hinshaw, 2016). ASD has also been associated with higher risk of physical (Dammeyer & Chapman, 2018; Ohlsson Gotby et al., 2018) and sexual victimization (Brown-Lavoie, Viecili, & Weiss, 2014; Dammeyer & Chapman, 2018; Ohlsson Gotby et al., 2018; Weiss & Fardella, 2018). Similar results on vulnerability to physical and sexual victimization have also been found for ID (Fogden, Thomas, Daffern, & Ogloff, 2016; Nixon, Thomas, Daffern, & Ogloff, 2017). However, most of the available evidence is based on cross-sectional data, with retrospective assessment of neurodevelopmental disorders or symptoms (Brown-Lavoie et al., 2014; Dammeyer & Chapman, 2018; McCauley et al., 2015; Weiss & Fardella, 2018; Wymbs, Dawson, Egan, & Sacchetti, 2019; Wymbs, Dawson, Suhr, Bunford, & Gidycz, 2017). This may limit the ability to establish the temporal order of the exposure and the outcome and raises the issue of recall bias.

A recent register-based study from Denmark used information on criminal victimization from police records and on psychiatric diagnoses from medical records, and found that having a diagnosis of ND was associated with a higher risk of being subjected to criminal victimization in females, but not in males (Dean et al., 2018). However, the associations attenuated when adjusting for other psychiatric disorders, family characteristics, and criminal offending, with the exception of ID in women, which still represented a risk factor for being victim of violence (Dean et al., 2018). Another study based on a survey among university students did not find an association between self-reported ADHD symptoms and physical intimate partner violence victimization (Wymbs et al., 2017). Hence, it remains unclear if all or just some NDs are associated with an increased risk of victimization and if the risk varies by sex. These are important knowledge gaps as violent victimization is associated with a range of negative outcomes, including depression, anxiety, post-traumatic stress disorder, substance use problems, self-harm, criminality, and violence perpetration (Latalova, Kamaradova, & Prasko, 2014; Turanovic & Pratt, 2015; Vaughn et al., 2010).

None of the previous studies examined whether shared familial factors, including genetics, may explain the association between NDs and victimization. For example, previous research has shown that genetic vulnerability to psychiatric disorders, including ADHD, is correlated with exposure to another type of victimization, bullying (Schoeler et al., 2019). Another relevant mechanism to explore is the role of criminal offending and other externalizing problems. For example, it is well established that ADHD is associated with an increased risk of criminality (Lichtenstein et al., 2012; Mohr-Jensen, Müller Bisgaard, Boldsen, & Steinhausen, 2019; Mohr-Jensen & Steinhausen, 2016) and substance use disorder (SUD) (Biederman et al., 1997; Groenman, Janssen, & Oosterlaan, 2017; Groenman et al., 2013; Yoshimasu et al., 2012), which, in turn, are associated with greater risk of being victim of violence (Johnson et al., 2016; Vaughn et al., 2010). As a result, SUD and criminality may mediate the association between NDs and violent victimization. Therefore, assessing the role of familial confounding and mediating factors may help clarifying the mechanisms through which NDs may influence the vulnerability to victimization in adolescence and early adulthood.

Taken together, previous studies suggest that risk of violent victimization should be considered among those with NDs. However, use of cross-sectional data and lack of consideration of important potential confounders and mediators may generate biased results. In this study, we investigated the association between several NDs and subsequent risk of severe violent victimization in adolescence and young adulthood, using prospectively collected data on hospitalizations and deaths due to assault. We had two main aims. First, we wanted to estimate the crude association between different NDs and victimization in males and females, in order to establish the extent to which different NDs were associated with risk of violent victimization and if there may be sex differences. This would allow identifying the most vulnerable patient groups. Second, we explored two mechanisms that may explain the association between NDs and victimization, that is, shared familial factors and mediation via externalizing problems. To do so, we examined whether the associations between NDs and victimization was explained by unmeasured shared familial effects by comparing violent victimization rates among sibling pairs who are discordant on their diagnosis status. This method uses information on pairs where one sibling is diagnosed with a ND and the other is not in order to adjust for familial factors shared by the siblings, such as their socio-economic background and half of their genetic makeup (D'Onofrio, Lahey, Turkheimer, & Lichtenstein, 2013). Furthermore, we tested whether the association between NDs and victimization was explained by externalizing problems, including conduct disorder (CD), SUD, and criminal convictions, which may account, at least in part, for the association between some NDs and violent victimization.

## Methods

The study was approved by the regional ethics review board in Stockholm, Sweden. The requirement for informed consent was waived because the study was register-based and it was not possible to identify the included individuals.

### Study population

We used data from a linkage of several national Swedish registers via the unique identification number (Ludvigsson, Otterblad-Olausson, Pettersson, & Ekbom, 2009). Using information from the Total Population Register (Ludvigsson et al., 2016), we included in the study all individuals born in Sweden between 1985 and 1997 alive and living in Sweden at their 15^th^ birthday, with identifiable biological parents. We followed them from their 15^th^ birthday until the event of interest, death, migration outside Sweden, or December 31^st^ 2013, whichever came first. That date would represent the end of the follow-up. We followed individuals from age 15 in order to focus on victimization events during late adolescence and early adulthood and we excluded individuals with a victimization event before age 15. Within the study population, we linked individuals with the same biological parents using both parents’ unique identification numbers to identify clusters of full-siblings.

### NDs

Using the National Patient Register (Ludvigsson et al., 2011), we identified diagnoses of NDs from specialist inpatient and outpatient visits after age two, using a similar classification as DSM-5, which includes ADHD, ASD, ID, communication disorders, specific learning disorder, motor disorders, and other/unspecified neurodevelopmental disorders. ADHD, ASD and ID, which were the most prevalent NDs in the sample, were also examined separately. A complete list of the International Classification of Diseases, 9^th^ revision (ICD-9), and ICD-10 codes is reported in Supplementary Table 1.

All the exposures were time-varying. Individuals who were diagnosed with NDs before the 15^th^ birthday were considered exposed for the entire follow-up. Individuals who were diagnosed with NDs after their 15^th^ birthday were considered as unexposed from their 15^th^ birthday until the first diagnosis, and as exposed after the first diagnosis.

### Violent victimization

Using information from the National Patient Register (Ludvigsson et al., 2011) and the Cause of Death Register (Brooke et al., 2017), we defined violent victimization as any inpatient or outpatient visit or death due to assault (ICD-9 codes: E960-E969; ICD-10 codes: X85-Y09), in line with previous studies (Sariaslan, Arseneault, Larsson, Lichtenstein, & Fazel, 2020; Sariaslan, Lichtenstein, Larsson, & Fazel, 2016). The outcome date was defined as the date of first registered diagnosis. As the National Patient Register includes data from inpatient and outpatient visits, these events may be considered quite severe events, as they were treated in specialist care. Primary care data are not included in the National Patient Register.

### Other covariates

The following covariates were considered: year of birth, using information from the Total Population Register (1985-1990;1991-1997); diagnosis of CD in the National Patient Register (ICD-9 code: 312; ICD-10 code: F91); diagnosis of SUD in the National Patient Register (ICD-9 codes: 291, 292, 303-305; ICD-10 code: F10-F19); crime conviction (Sariaslan et al., 2020, Sariaslan et al., 2016), identified in the National Crime Register, which includes information on all criminal convictions in Sweden since 1973 for individuals aged 15 or older, which is the age of criminal responsibility in Sweden.

### Statistical analysis

We conducted all analyses separately for males and females, as NDs have different prevalence in males and females and results from previous studies suggest that there may be sex differences in the vulnerability to different types of victimizations.

First, to explore the crude association between the NDs and violent victimization among males and females, we plotted the estimated cumulative incidence of being violently victimized in exposed and unexposed groups by sex using the Kaplan–Meier method. Then, we used Cox regression model to estimate the hazard ratios (HR) and 95% confidence intervals (CIs) for time to violent victimization, with cluster-robust standard errors accounting for the correlated data from full-siblings. The underlying time scale was time since the start of the follow-up, that is, the 15^th^ birthday. We performed the analysis for all NDs combined, as well as for ADHD, ASD and ID separately and then with ADHD, ASD, and ID included together in a multiple regression model. Therefore, estimates for each disorder from this model were adjusted for the other disorders. This was done in order to establish if all NDs or only some disorders were uniquely associated with risk of violent victimization. In addition, we evaluated if there was any difference between having only one disorder vs having more than one. In order to do so, we considered ADHD, ASD, and ID in separate models as exposures on three different levels: no diagnosis, diagnosis of one disorder, diagnosis of more than one disorder. That is, when considering ADHD, one could have no diagnosis of ADHD, only a diagnosis of ADHD, or a diagnosis of ADHD and of ASD and/or ID. When considering ASD, one could have no diagnosis of ASD, only a diagnosis of ASD, or a diagnosis of ASD and ADHD and/or ID. When considering ID, one could have no diagnosis of ID, only a diagnosis of ID, or a diagnosis of ID and of ADHD and/or ASD.

Second, we used stratified Cox regression model entering each cluster of full-siblings as a separate stratum (model adjusted for familial factors). This approach allows adjusting for all potential confounders that are constant within each cluster of siblings during the follow-up. Only clusters of siblings with variation in at least one of the covariates and at least one outcome event contribute to this analysis (Number of clusters=26,337; Number of individuals=54,354). In this model, we also adjusted for year of birth to control for potential temporal trends, which may for example affect administrative prevalence of NDs. This was done in order to explore if shared familial factors would explain potential associations between NDs and violent victimization.

Third, we added the following externalizing problems to the stratified Cox regression model of full-siblings (that is, the model adjusted for familial factors explained above): diagnosis of SUD, diagnosis of CD, crime conviction (model adjusted for familial factors and mediators). These covariates were time varying and date of the first diagnosis or first crime was used as the starting date of the exposed time. Hence, individuals who were diagnosed with SUD or CD before or at the 15^th^ birthday were considered exposed for the entire follow-up, while individuals who were diagnosed with SUD or CD after their 15^th^ birthday were considered as unexposed from their 15^th^ birthday until the first diagnosis, and as exposed afterwards. This was done in order to explore if externalizing problems would explain potential associations between NDs and violent victimization.

As a sensitivity analysis, we conducted these analyses considering ND diagnoses after age four, for a more conservative definition of the disorders.

## Results

### Description of the sample

Descriptive statistics of the study population, which included 1,344,944 individuals, are reported in Table 1. More than five percent of the study population (N=74,487; 5.54%) were diagnosed with a ND, of which ADHD was the most common (N=45,991; 3.42%). During the follow-up (mean length=5 years; average age at the end of the follow-up=23), 37,765 (2.81%) individuals who were violently victimized, and males (N=26,884; 3.90%) were more at risk than females (N=10,881; 1.66%).

### Crude associations between NDs and violent victimization

**Figure 1:**
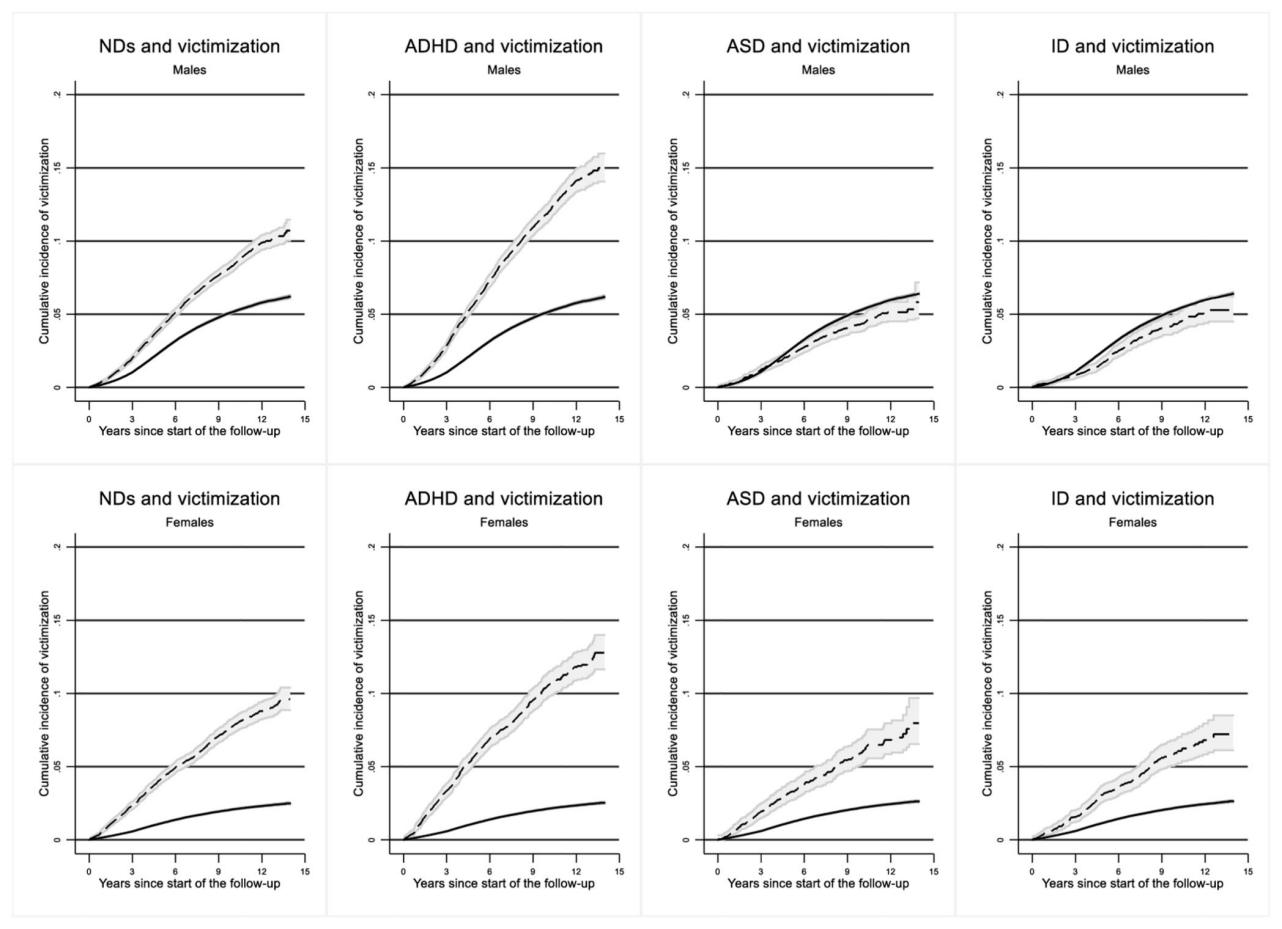
Cumulative incidence of violent victimization Note: Dashed line=exposed to NDs; solid line=unexposed to NDs. Abbreviation: NDs=neurodevelopmental disorders; ASD=autism spectrum disorder; ID=intellectual disability.

Figure 1 depicts cumulative incidence of violent victimization. At the end of the follow-up, the estimated cumulative incidence of violent victimization after being diagnosed with any NDs was 10.8% (95% CI=10.3-11.4) in males and 9.7% (95% CI=9.0-10.5) in females, compared to 6.2% (95% CI=6.1-6.3) in males and 2.4% (95% CI=2.4-2.5) in females not diagnosed with NDs. The difference was larger for ADHD, with an estimated cumulative incidence of violent victimization equal to 16.0% (95% CI=15.0-17.0) in males and 13.2% (95% CI=12.1-14.3) in females diagnosed with ADHD, compared to 6.2% (95% CI=6.1-6.2) in males and 2.5% (95% CI=2.4-2.5) in females not diagnosed with ADHD.

Crude associations between NDs and violent victimization are reported in Table 2. A diagnosis of ND was associated with an increased risk of subsequent violent victimization in males (HR=1.72; 95% CI=1.64-1.80) and females (HR=3.94; 95% CI=3.68-4.22). Among males, when considering specific disorders in separate models, only ADHD was associated with an increased risk of violent victimization (HR=2.56; 95% CI=2.43-2.70), while ASD and ID were associated with a reduced risk of violent victimization. Among females, all NDs were associated with an increased risk of violent victimization. When considering ADHD, ASD, ID simultaneously in a multiple regression model, among males, only ADHD was associated with an increased risk of violent victimization, while ASD and ID were associated with a reduced risk of violent victimization. In contrast, among females, all disorders were independently associated with an increased risk of violent victimization, with a stronger association for ADHD (HR=4.91; 95% CI=4.48-5.38) than for ASD (HR=1.24; 95% CI=1.04-1.47) and ID (HR=1.77; 95% CI=1.51-2.07). The difference in the association between NDs and violent victimization between males and females was statistically significant (p=0.00).

When evaluating the role of comorbidity, the pattern of results for ADHD was different from ASD and ID. For ADHD, among males, having only a diagnosis of ADHD was associated with an increased risk of violent victimization (HR=2.86; 95% CI=2.70-3.03), as compared to not having a diagnosis of ADHD. The increase in the risk was lower if there was an additional diagnosis of ASD and/or ID (HR=1.61; 95% CI=1.42-1.85). Among females, having only a diagnosis of ADHD (HR=5.45; 95% CI=4.98-5.97) or having an additional diagnosis of ASD and/or ID (HR=5.15; 95% CI=4.32-6.15) was associated with an increase in the risk of violent victimization of similar magnitude. For ASD, among males, having only a diagnosis of ASD was associated with a decreased risk of violent victimization (HR=0.74; 95% CI=0.62-0.88). The association for having an additional diagnosis of ADHD and/or ID was null (HR=0.96; 95% CI=0.81-1.13). Among females, having only a diagnosis of ASD (HR=2.71; 95% CI=2.20-3.33) or having an additional diagnosis of ADHD and/or ID (HR=3.04; 95% CI=2.47-3.75) was associated with an increase in the risk of violent victimization of similar magnitude. For ID, among males, having only a diagnosis of ID was associated with a decreased risk of violent victimization HR=0.63 (95% CI=0.51-0.78). The association for having an additional diagnosis of ADHD and/or ASD was null (HR=1.01 (95% CI=0.83-1.21). Among females, having only a diagnosis of ID (HR=2.50; 95% CI=2.08-2.99) or having an additional diagnosis of ADHD and/or ASD (HR=3.41; 95% CI=2.71-4.31) was associated with an increase in the risk of violent victimization of similar magnitude.

### The role of familial factors for associations between NDs and violent victimization

Adjusted associations between NDs and violent victimization are reported in Table 3. In the model where we explored to role of familial factors shared by full-siblings, a diagnosis of ND was associated with an increased risk of subsequent violent victimization in males (HR=1.14; 95% CI=0.99-1.31), although the confidence interval included one, and in females (HR=1.73; 95% CI=1.37-2.18). All familial factor adjusted estimates (Table 3) attenuated compared to the non-adjusted estimates (Table 2), which suggests that familial factors may explain part of the association. When considering the specific disorders simultaneously, only ADHD was associated with an increased risk of violent victimization in both males (HR=1.53; 95% CI=1.29-1.82) and females (HR=2.24; 95% CI=1.64-3.04).

### The role of mediators for associations between NDs and violent victimization

The associations further attenuated when considering the role of familial factors and externalizing problems, suggesting that these may be important mechanisms in the association between NDs and violent victimization (Table 3). When examining specific disorders, only ADHD was associated with an increased risk of violent victimization, both in males (HR=1.27; 95% CI=1.06-1.51) and in females (HR=1.69; 95% CI=1.21-2.36; Table 3).

### Sensitivity analysis

Associations between a more conservative definition of NDs and violent victimization were similar to those obtained from the main analyses (Table 4).

## Discussion

In this nation-wide study, we found that having a diagnosis of NDs was associated with an increased risk of being victim of violence during adolescence and young adulthood among females. In males, there was a positive association with ADHD only, while ASD and ID were associated with a decreased risk of violent victimization. A possible explanation for this is that ADHD symptoms, such as impulsivity and inattention, may increase the vulnerability of both men and women to violent victimization. In contrast, ASD symptoms, such as social and communication difficulties, may have opposite effects in males and females. For example, young women might become the target of violence, because of their interpersonal difficulties, whereas having fewer interactions with others or even being socially isolated may protect young men from contexts where violence may occur. When considering the role of comorbidities, we found that among males with ADHD, having and additional diagnosis of ASD, ID or both, may reduce the magnitude of the increase in the risk of violent victimization. In other words, comorbidity with these disorders may be protective. Among females, for any of the disorders considered comorbidity did not seem to result in a larger increase in the risk of violent victimization.

The analyses of the possible mechanisms underlying the observed associations revealed three important findings. First, sibling-comparisons led to attenuated associations, indicating that the mechanisms underlying associations between NDs and violent victimization reflect in part a shared familial liability. This is in line with a recent Swedish study on the risk of violent victimization among individuals with other psychiatric disorders, which found increased risk for all psychiatric disorders, but attenuated estimates in models adjusted for familial confounding using sibling-comparison design (Sariaslan et al., 2020). This is also in line with evidence suggesting that genetic vulnerability to psychiatric disorders is associated with an increased risk of being exposed to a less severe form of victimization, bullying (Schoeler et al., 2019).

Second, the disorder-specific sibling-comparisons (that is, the model adjusted for familial factors by comparing siblings who are discordant on diagnosis of NDs) revealed that the association of ASD with violent victimization in females was mostly attenuated to one, while ADHD remained associated with an increased risk of violent victimization in men and women separately. This suggests that, although part of the association was explained by shared familial factors, ADHD may be independently associated with a higher risk of being violently victimized. One possible explanation for this is that ADHD symptoms such as impulsivity and/or reduced vigilance (i.e., inattention) to potential threats may be increase the risk of becoming victim of violence.

Third, we found that the association between ADHD and victimization was partly mediated by externalizing behaviours. This is consistent with the well-established association between ADHD and externalizing problems (Biederman et al., 1997; Groenman et al., 2017; Groenman et al., 2013; Lichtenstein et al., 2012; Mohr-Jensen et al., 2019; Mohr-Jensen & Steinhausen, 2016; Yoshimasu et al., 2012) and the available evidence supporting an association between externalizing problems and risk of becoming victim of violence (Johnson et al., 2016; Vaughn et al., 2010). In addition, these findings confirm the importance of the dynamic interplay between victimization and perpetration risk (Sariaslan et al., 2020), which needs to be considered carefully in future research. Importantly, the association between ADHD and violent victimization was not fully explained by externalizing problems. This suggests that impulsivity and lack of attention to potentially dangerous situations may be important factors influencing the risk of being victim of violence, even among individuals who do not have a history of conduct, abuse, or crime issues. Future research may explore symptom domains and mechanisms that may underlie this association and design interventions to target them.

Results from this study are in line with previous research, which has found an overall increased risk of victimization of different type and severity among individuals with NDs, using data from surveys (Brown-Lavoie et al., 2014; Dammeyer & Chapman, 2018; Fogden et al., 2016; Guendelman et al., 2016; McCauley, Breslau, Saito, & Miller, 2015; Nixon et al., 2017; Ohlsson Gotby et al., 2018; Weiss & Fardella, 2018; Wymbs et al., 2019). Considering previous and current evidence, mental health professionals should carefully consider the risk of individuals with ND of being victim of violence and help patients and their families to identify situations or behaviours that may be unsafe. However, these results are partially in contrast to what has been reported by a recent Danish study, which found that, after several adjustments, a diagnosis of ND was not associated with a higher risk of violent victimization, with the exception of women with ID (Dean et al., 2018). The divergence in results may be explained by methodological differences, including the choice of not considering different NDs separately and the use of data on police-reported crimes. In fact, different data sources may capture different types of events. For example, hospital and death records capture only victimization events severe enough to require medical attention.

A number of limitations should be considered when interpreting the results of the study. First, as mentioned above, we only had data on clinical diagnoses of NDs and violent victimization from medical and death records, which capture mainly more severe cases, both for NDs and for violent victimization. Therefore, our results might not generalize to less severe ND symptoms and victimization events. In addition, it may be that individuals with different NDs may be more or less likely to report violence, due to the symptoms of the disorders. For example, communication difficulties typical of ASD may influence the likelihood of seeking medical care or reporting being victim of violence. Second, we could not investigate which symptoms of NDs (e.g., for ADHD impulsivity vs. inattentiveness) may be more strongly associated with the risk of victimization because we did not have access to information on disorder subtypes or manifestations, or symptom dimensions. Similarly, we could not differentiate between different types of victimization events, as more detailed data are not be always available or accurate. Third, data on CD, SUD, and crime conviction may not be an optimal measure of externalizing behaviours, due to underreporting. If this was the case, the independent association between ADHD and violent victimization may be overestimated. Future studies, with more comprehensive assessment of externalizing problems, may help to elucidate the interplay between ADHD, externalizing problems and victimization. In addition, although we interpreted the role of externalizing problems as possible mediators of the association between NDs and victimization, we did not restrict the timing of externalizing problems (that is either the diagnosis of SUD or CD or crime conviction) to occur after ND diagnoses, in order to use all the available information from the different registers. This was done because while it may happen that, for example, a diagnosis of ADHD is given after a SUD diagnosis, this is likely to reflect a delayed diagnosis of ADHD, as ADHD typically has its onset in childhood, while SUD typically has its onset in adolescence or early adulthood. In addition, childhood ADHD is recognized as a risk factor for subsequent SUD (Biederman et al., 1997; Groenman et al., 2017; Groenman et al., 2013; Yoshimasu et al., 2012). Fourth, although we excluded individuals who had a victimization event before the start of the follow-up, there might be unrecorded events due to, for example, later start of the outpatient register (in 2001) and this may be an issue for the older individuals in the cohort. In addition, we were not able to control for other less severe events not recorded in healthcare registers, which may be confounders of the association between NDs and violent victimization, as they may be associated with an increased probability of both. While such events may be accounted for in the analysis based on sibling-comparison, that is, adjusted for familial factors shared by siblings, we cannot exclude that such factors may also act at the individual level. Last, sibling-comparison only accounts for part of the genetic influences, since siblings share on average half of their co-segregating alleles. Therefore, residual genetic confounding may explain at least part of the association between ADHD and violent victimization.

In conclusion, ADHD, but also ASD and ID in females, are associated with increased risk of violent victimization throughout adolescence and emerging adulthood. Therefore, mental health professionals should take into consideration the vulnerability of these patients to being victim of severe violence and ask about victimization experiences, in order to provide appropriate support and prevent secondary negative effects. The mechanisms explaining the observed associations reflect in part a shared familial liability, but also mediation via externalizing problems. In addition, for ADHD, there might be an independent association with violent victimization, which deserves further investigation to clarify relevant symptom dimensions and mechanisms.

## Supplementary Material

Table S1

## Figures and Tables

**Table 1:** Description of the study population

	Overall	Males	Females
	No. (%)	No. (%)	No. (%)
Whole study population	1,344,944	689,878 (51.29)	655,066 (48.71)
Any ND	74,487 (5.54)	46,603 (6.76)	27,884 (4.26)
ADHD	45,991 (3.42)	28,899 (4.19)	17,092 (2.61)
ASD	21,362 (1.59)	14,058 (2.04)	7,304 (1.12)
ID	14,194 (1.06)	8,400 (1.22)	5,794 (0.88)
SUD	59,886 (4.45)	32,029 (4.64)	27,857 (4.25)
CD	5,327 (0.40)	3,310 (0.48)	2,017 (0.31)
Any crime conviction	160,606 (11.94)	114,666 (16.62)	45,940 (7.01)
Maternal psychiatric diagnosis	206,563 (15.36)	106,476 (15.43)	100,087 (15.28)
Paternal psychiatric diagnosis	175,093 (13.02)	89,400 (12.96)	85,693 (13.08)
Maternal upper secondary education^a^	1,176,827 (89.46)	603,517 (89.47)	573,310 (89.45)
Paternal upper secondary education^b^	1,049,921 (82.40)	538,843 (82.42)	511,078 (82.38)
Violent victimization event	37,765 (2.81)	26,884 (3.90)	10,881 (1.66)

Abbreviation: No.=number of observations; ND=neurodevelopmental disorder; ADHD=attention-deficit/hyperactivity disorder; ASD=autism spectrum disorder; ID=intellectual disability; SUD=substance use disorder; CD=conduct disorder.

a Note: due to missing values, N=1,315,490;

b due to missing values, N=1,298,388.

**Table 2:** Crude associations between neurodevelopmental disorders and violent victimization

	Separate models	Multiple regression model^a^
	HR (95% CI)	HR (95% CI)
**Males**		
Any ND	1.72 (1.64-1.80)	NA
ADHD	2.56 (2.43-2.70)	2.83 (2.67- 2.99)
ASD	0.84 (0.75-0.95)	0.60 (0.53-0.68)
ID	0.80 (0.70-0.92)	0.65 (0.56-0.75)
**Females**		
Any ND	3.94 (3.68-4.22)	NA
ADHD	5.39 (4.97-5.85)	4.91 (4.48-5.38)
ASD	2.87 (2.47-3.32)	1.24 (1.04-1.47)
ID	2.78 (2.41-3.20)	1.77 (1.51-2.07)

Abbreviation: HR=hazard ratio; CI=confidence interval; ND=neurodevelopmental disorder; ADHD=attention-deficit/hyperactivity disorder; ASD=autism spectrum disorder; ID=intellectual disability.

a the estimates for each disorder are mutually adjusted for the other disorders.

**Table 3:** Adjusted associations between neurodevelopmental disorders and violent victimization

	Adjusted for familial factors^a^	Adjusted for familial factors and mediators^b^
	HR (95% CI)	HR (95% CI)
**Males**		
Any ND	1.14 (0.99-1.31)	0.99 (0.86-1.14)
ADHD	1.53 (1.29-1.82)	1.27 (1.06-1.51)
ASD	0.82 (0.59-1.12)	0.83 (0.61-1.13)
ID	0.49 (0.34-0.70)	0.51 (0.36-0.73)
**Females**		
Any ND	1.73 (1.37-2.18)	1.42 (1.11-1.83)
ADHD	2.24 (1.64-3.04)	1.69 (1.21-2.36)
ASD	0.81 (0.49-1.34)	0.75 (0.45-1.2 4)
ID	1.29 (0.80-2.11)	1.29 (0.80-2.08)

Abbreviation: HR=hazard ratio; CI=confidence interval; ND=neurodevelopmental disorder; ADHD=attention-deficit/hyperactivity disorder; ASD=autism spectrum disorder; ID=intellectual disability.

a Notes: the model is adjusted for familial factors shared by full siblings;

b the model is additionally adjusted for substance use disorder, conduct disorder, and crime conviction.

**Table 4:** Sensitivity analysis on neurodevelopmental disorder diagnoses from age four

	Crude association –Separate models	Crude association –Multiple regression model^a^	Adjusted for familial factors^b^	Adjusted for familial factors and mediators^c^
	HR (95% CI)	HR (95% CI)	HR (95% CI)	HR (95% CI)
**Males**				
Any ND	1.74 (1.66-1.83)	NA	1.16 (1.01-1.34)	1.01 (0.87-1.17)
ADHD	2.56 (2.43-2.71)	2.83 (2.67-2.99)	1.52 (1.28-1.80)	1.25 (1.05-1.49)
ASD	0.86 (0.76-0.96)	0.60 (0.53-0.68)	0.83 (0.60-1.15)	0.85 (0.62-1.16)
ID	0.81 (0.71-0.94)	0.66 (0.57-0.76)	0.50 (0.35-0.71)	0.52 (0.37-0.74)
**Females**				
Any ND	3.84 (3.71-4.25)	NA	1.73 (1.37-2.19)	1.43 (1.11-1.84)
ADHD	5.40 (5.00-5.86)	4.93 (4.50-5.40)	2.24 (1.65-3.06)	1.69 (1.21-2.37)
ASD	2.85 (2.45-3.30)	1.22 (1.03-1.45)	0.79 (0.47-1.31)	0.73 (0.44-1.21)
ID	2.81 (2.43-3.24)	1.79 (1.53-2.09)	1.26 (0.77-2.06)	1.26 (0.78-2.03)

Abbreviation: HR=hazard ratio; CI=confidence interval; ND=neurodevelopmental disorder; ADHD=attention-deficit/hyperactivity disorder; ASD=autism spectrum disorder; ID=intellectual disability.

a the estimates for each disorder are mutually adjusted for the other disorders;

b the model is adjusted for familial factors shared by full siblings;

c the model is additionally adjusted for substance use disorder, conduct disorder, and crime conviction.

## References

[R1] APA (2013). Diagnostic and statistical manual of mental disorders (DSM-5®).

[R2] Biederman J, Wilens T, Mick E, Faraone SV, Weber W, Curtis S, Thornell A, Pfister K, Jetton JG, Soriano J (1997). Is ADHD a risk factor for psychoactive substance use disorders? Findings from a four-year prospective follow-up study. Journal of the American Academy of Child and Adolescent Psychiatry.

[R3] Brooke HL, Talbäck M, Hörnblad J, Johansson LA, Ludvigsson JF, Druid H, Feychting M, Ljung R (2017). The Swedish cause of death register. European journal of epidemiology.

[R4] Brown-Lavoie SM, Viecili MA, Weiss JA (2014). Sexual knowledge and victimization in adults with autism spectrum disorders. Journal of Autism and Developmental Disorders.

[R5] D'Onofrio BM, Lahey BB, Turkheimer E, Lichtenstein P (2013). Critical need for family-based, quasi-experimental designs in integrating genetic and social science research. American Journal of Public Health.

[R6] Dammeyer J, Chapman M (2018). A national survey on violence and discrimination among people with disabilities. BMC Public Health.

[R7] Dean K, Laursen TM, Pedersen CB, Webb RT, Mortensen PB, Agerbo E (2018). Risk of Being Subjected to Crime, Including Violent Crime, After Onset of Mental Illness: A Danish National Registry Study Using Police Data. JAMA Psychiatry.

[R8] Fogden BC, Thomas SD, Daffern M, Ogloff JR (2016). Crime and victimisation in people with intellectual disability: a case linkage study. BMC Psychiatry.

[R9] Groenman AP, Janssen TWP, Oosterlaan J (2017). Childhood Psychiatric Disorders as Risk Factor for Subsequent Substance Abuse: A Meta-Analysis. Journal of the American Academy of Child and Adolescent Psychiatry.

[R10] Groenman AP, Oosterlaan J, Rommelse N, Franke B, Roeyers H, Oades RD, Sergeant JA, Buitelaar JK, Faraone SV (2013). Substance use disorders in adolescents with attention deficit hyperactivity disorder: a 4-year follow-up study. Addiction.

[R11] Guendelman MD, Ahmad S, Meza JI, Owens EB, Hinshaw SP (2016). Childhood Attention-Deficit/Hyperactivity Disorder Predicts Intimate Partner Victimization in Young Women. Journal of Abnormal Child Psychology.

[R12] Johnson KL, Desmarais SL, Tueller SJ, Grimm KJ, Swartz MS, Van Dorn RA (2016). A longitudinal analysis of the overlap between violence and victimization among adults with mental illnesses. Psychiatry Research.

[R13] Latalova K, Kamaradova D, Prasko J (2014). Violent victimization of adult patients with severe mental illness: a systematic review. Neuropsychiatric Disease and Treatment.

[R14] Lichtenstein P, Halldner L, Zetterqvist J, Sjolander A, Serlachius E, Fazel S, Langstrom N, Larsson H (2012). Medication for attention deficit-hyperactivity disorder and criminality. New England Journal of Medicine.

[R15] Ludvigsson JF, Almqvist C, Bonamy A-KE, Ljung R, Michaëlsson K, Neovius M, Stephansson O, Ye W (2016). Registers of the Swedish total population and their use in medical research. European Journal of Epidemiology.

[R16] Ludvigsson JF, Andersson E, Ekbom A, Feychting M, Kim J-L, Reuterwall C, Heurgren M, Olausson PO (2011). External review and validation of the Swedish national inpatient register. BMC Public Health.

[R17] Ludvigsson JF, Otterblad-Olausson P, Pettersson BU, Ekbom A (2009). The Swedish personal identity number: possibilities and pitfalls in healthcare and medical research. European Journal of Epidemiology.

[R18] McCauley HL, Breslau JA, Saito N, Miller E (2015). Psychiatric disorders prior to dating initiation and physical dating violence before age 21: findings from the National Comorbidity Survey Replication (NCS-R). Social Psychiatry and Psychiatric Epidemiology.

[R19] Mohr-Jensen C, Müller Bisgaard C, Boldsen SK, Steinhausen HC (2019). Attention-Deficit/Hyperactivity Disorder in Childhood and Adolescence and the Risk of Crime in Young Adulthood in a Danish Nationwide Study. Journal of the American Academy of Child and Adolescent Psychiatry.

[R20] Mohr-Jensen C, Steinhausen H-C (2016). A meta-analysis and systematic review of the risks associated with childhood attention-deficit hyperactivity disorder on long-term outcome of arrests, convictions, and incarcerations. Clinical Psychology Review.

[R21] Nixon M, Thomas SDM, Daffern M, Ogloff JRP (2017). Estimating the risk of crime and victimisation in people with intellectual disability: a data-linkage study. Social Psychiatry and Psychiatric Epidemiology.

[R22] Ohlsson Gotby V, Lichtenstein P, Langstrom N, Pettersson E (2018). Childhood neurodevelopmental disorders and risk of coercive sexual victimization in childhood and adolescence - a population-based prospective twin study. The Journal of Child Psychology and Psychiatry.

[R23] Sariaslan A, Arseneault L, Larsson H, Lichtenstein P, Fazel S (2020). Risk of Subjection to Violence and Perpetration of Violence in Persons With Psychiatric Disorders in Sweden. JAMA Psychiatry.

[R24] Sariaslan A, Lichtenstein P, Larsson H, Fazel S (2016). Triggers for Violent Criminality in Patients With Psychotic Disorders. JAMA Psychiatry.

[R25] Schoeler T, Choi SW, Dudbridge F, Baldwin J, Duncan L, Cecil CM, Walton E, Viding E, McCrory E, Pingault JB (2019). Multi-Polygenic Score Approach to Identifying Individual Vulnerabilities Associated With the Risk of Exposure to Bullying. JAMA Psychiatry.

[R26] Turanovic JJ, Pratt TC (2015). Longitudinal effects of violent victimization during adolescence on adverse outcomes in adulthood: a focus on prosocial attachments. Journal of Pediatrics.

[R27] Vaughn MG, Fu Q, DeLisi M, Beaver KM, Perron BE, Howard MO (2010). Criminal victimization and comorbid substance use and psychiatric disorders in the United States: results from the NESARC. Annals of Epidemioliogy.

[R28] Weiss JA, Fardella MA (2018). Victimization and Perpetration Experiences of Adults With Autism. Frontiers in Psychiatry.

[R29] Wymbs BT, Dawson AE, Egan TE, Sacchetti GM (2019). Rates of Intimate Partner Violence Perpetration and Victimization Among Adults With ADHD. Journal of attention disorders.

[R30] Wymbs BT, Dawson AE, Suhr JA, Bunford N, Gidycz CA (2017). ADHD Symptoms as Risk Factors for Intimate Partner Violence Perpetration and Victimization. Journal of Interpersonal Violence.

[R31] Yoshimasu K, Barbaresi WJ, Colligan RC, Voigt RG, Killian JM, Weaver AL, Katusic SK (2012). Childhood ADHD is strongly associated with a broad range of psychiatric disorders during adolescence: a population-based birth cohort study. The Journal of Child Psychology and Psychiatry.

