# Supplementary material for: Neurodevelopmental disorders and subsequent risk of violent victimization: exploring sex differences and mechanisms
Source: Psychol Med. Author manuscript; Available in PMC 2025 Oct 10. (PMC7618236; doi:10.1017/S0033291721003093)
Supplement: Table S1 [file EMS208264-supplement-Table_S1.docx]

**Neurodevelopmental disorders and subsequent risk of violent victimization: exploring sex differences and mechanisms**

Laura Ghirardi, PhD^1^; Ralf Kuja-Halkola, PhD^1^; Erik Pettersson, PhD^1^; Amir Sariaslan, PhD;^2^ Louise Arseneault, PhD;^3^ Seena Fazel, MBChB, MD^4^; Brian M. D’Onofrio, PhD;^1,5^ Paul Lichtenstein, PhD;^1^ Henrik Larsson, PhD. ^1,6^

1 Department of Medical Epidemiology and Biostatistics, Karolinska Institutet, Stockholm, Sweden

2 Faculty of Social Sciences, Social and Public Policy Unit, University of Helsinki, Helsinki, Finland

3 Social, Genetic, and Developmental Psychiatry Centre, Institute of Psychiatry, Psychology, and Neuroscience, King’s College London, London, United Kingdom

4 Department of Psychiatry, University of Oxford, Warneford Hospital, Oxford, United Kingdom

5 Department of Psychological and Brain Sciences, Indiana University, Bloomington, Indiana

6 School of Medical Sciences, Örebro University, Örebro, Sweden

**Supplementary table 1. ICD-9 and ICD-10 codes for neurodevelopmental disorders**

| **Category (DSM-5)** | **ICD-9 codes** | **ICD-10 codes** |
| --- | --- | --- |
| ADHD | 314 | F90 |
| ASD | 299 | F84 |
| ID | 317-319 | F70-F73; F78-F79 |
| Other neurodevelopmental disorders (e.g: communication disorders, learning disorders, motor disorders) | 307.2, 307.3, 315 | F80, F81, F82, F88, F89, F984, F95, R48 |

Abbreviations: NDs=neurodevelopmental disorders; ADHD=attention-deficit/hyperactivity disorder; ASD=autism spectrum disorder; ID=intellectual disability; DSM-5=Diagnostic and Statistical Manual of Mental Disorders, Fifth Edition; ICD-9=The International Classification of Diseases, Ninth Revision; ICD-10=The International Classification of Diseases, Tenth Revision.
